# Self-Regulation and Cognitive Agility in Cyber Operations

**DOI:** 10.3389/fpsyg.2019.00875

**Published:** 2019-04-24

**Authors:** Øyvind Jøsok, Ricardo Lugo, Benjamin James Knox, Stefan Sütterlin, Kirsi Helkala

**Affiliations:** ^1^Norwegian Defence Cyber Academy, Lillehammer, Norway; ^2^Faculty of Social and Health Sciences, Inland University of Applied Sciences, Lillehammer, Norway; ^3^Inland School of Business and Social Sciences, Inland University of Applied Sciences, Lillehammer, Norway; ^4^Department of Information Security and Communications Technology, Norwegian University of Science and Technology, Trondheim, Norway; ^5^Faculty for Health and Welfare Sciences, Østfold University College, Halden, Norway; ^6^Division of Clinical Neuroscience, Oslo University Hospital, Oslo, Norway

**Keywords:** self-regulation, cyber domain, cyber operations, defense, competence, cognitive agility

## Abstract

Reliance upon data networks to conduct military operations presents new challenges to the competence profiles of military personnel. Specifically the increased demand for the new category of military cyber personnel is a direct consequence of the utility of the cyber domain in contemporary military operations, both to support leadership processes and as a domain of operations on its own. The conflation of the cyber and physical domains empowers cyber operators to influence events beyond their immediate physical environment. Proper education and training of such personnel requires new insight into the competencies that are beyond cyber specific technical skills, to govern the complexity of operating in a cyber-physical hybrid environment. This pilot research contributes to the debate on military cyber personnel competencies by investigating how cyber defense operator’s level of self-regulation can contribute to their performance in operations. We hypothesize that higher levels of self-regulation predicts higher levels of cognitive agility as measured by cognitive movement in The Hybrid Space conceptual framework. Displays of cognitive agility within The Hybrid Space have previously been linked to performance in defensive cyber operations. A positive association was therefore expected between levels of self-regulation and displays of cognitive agility. *N* = 23 cyber cadets from the Norwegian Defence Cyber Academy (NDCA) completed self-regulation questionnaires (SRQs) and self-reported their cognitive location in The Hybrid Space during a 4-day cyber defense exercise. Data showed that higher levels of self-regulation were associated with displays of cognitive agility. According to the regression models in use, self-regulation could explain 43.1% of the total cognitive movements in The Hybrid Space. Understanding factors that contribute to cyber operator performance are needed to improve education and training programs for military cyber personnel. Validating self-regulation as a contributing factor to cognitive agility is important as this can be a pathway to empirically underpin individual cyber operator performance.

## Introduction

The increased utility of, and reliance upon, the cyber domain in military operations has led to higher demand of technically qualified cyber personnel (Champion et al., 2014). This is demonstrated through investment in cyber defense units, cyber defense education (NATO, 2016a), and the recognition of cyberspace as a domain of operations (NATO, 2016b). However, cyber operator tasks, competence requirements, and performance are unsettled concepts that lack clear definition and guidelines to support selection, education, and training of this new category military personnel. While technical cyber competence is paramount to operate in the cyber domain, the soft skills and cognitive competencies have started to receive more attention. The high cognitive demands of cyber operators have been widely acknowledged (Tapscott, 2014; Røislien, 2015; D’Amico et al., 2016; Buchler et al., 2018); however, the soft skills^1^ and cognitive competencies^2^ contribution to cyber operator performance is yet to be empirically validated (Forsythe et al., 2013; Lathrop et al., 2016; Helkala et al., 2017; Knox B. et al., 2018).

The Hybrid Space conceptual framework describes the hybrid character of the work environment of a military cyber operator and defines the cognitive space available for agile maneuver (Jøsok et al., 2016). The Hybrid Space framework theorizes that technical skills alone are not enough to perform in an age of network enabled operations (Buchler et al., 2016; Jøsok et al., 2016). The Hybrid Space framework acknowledges that the work environment of military cyber operators is influenced by factors like, e.g., team-work, leadership, hierarchy, communication, etc., but is also influenced by the intangible character of the digital context and information domain – consequently “shifting demands from physical fitness toward cognitive performance” (Knox B.J. et al., 2018, p. 351). It also allows the cyber operator to engage in strategic thinking while performing cyber operator tasks on a tactical level (Jøsok et al., 2016).

Some recent research contributions are addressing the cognitive competencies of cyber operators. Lathrop et al. (2016) propose that cyber operators are reliant on competencies like sensemaking, creative thinking, mental projection, and other high-level cognitive functions to perform. Further, cyber operators’ ability to collaborate, organize, and analyze problems has been described as: “… just as important as their technical acumen on the keyboard” (Buchler et al., 2018). However, it is unclear how these competencies relate to cyber operator performance. Knox B.J. et al. (2018) use The Hybrid Space framework to describe that individuals need to use different cognitive competencies to maneuver in The Hybrid Space. Examples include social-cognitive perspective-taking, spatial cognition, cognitive flexibility, macrocognition, metacognition, and self-regulation (Knox B.J. et al., 2018). The Hybrid Space framework has also previously been used to assess cyber operator cognitive agility during a cyber defense exercise. By utilizing the Hybrid Space framework, Knox et al. (2017) proposed cognitive agility as one important cognitive competency that could support cyber operator performance. They defined cognitive agility as “cognitive focus movements” in The Hybrid Space and later they associate displays of cognitive agility in The Hybrid Space with metacognition and performance of cyber operators (Knox et al., 2017). Metacognition is defined as “cognition of cognition” and is usually conceived as “an individual and conscious process that serves the regulation of cognition” (Efklides, 2008, p. 277). Self-regulation, a related concept, is defined as the regulation of cognition, emotions, behavior, and environment and includes metacognition in the process (Efklides, 2008). Self-regulation is a well-researched concept that has been shown to contribute to performance in other domains such as sport (Toering et al., 2009) and academic achievement (Zimmerman, 1990), but is yet to be researched in the military cyber operator context. In this article, we contribute to cyber operator competence profiles by investigating if cyber operators’ self-regulation is associated with performance in cyber operations. The authors hypothesize that higher levels of self-regulation predict cognitive agility as measured by cognitive movement in The Hybrid Space conceptual framework.

## Cyber Operator Cognitive Demands and Performance

The tasks in which cyber operators engage have been described as varied, often non-routine, and involve perception and comprehending large amounts of information (Erbacher et al., 2010). Cyber operator tasks include both human and technical aspects and: “…is heavily reliant upon the decision-making capabilities and skill-sets of defenders to overcome attackers” (Buchler et al., 2018). Ben-Asher and Gonzalez (2015) propose that cyber operators need updated theoretical knowledge, practical experience and training in how to: “…quickly learn and adapt to novel and dynamic environments” (p. 60). In addition, they address the need for this knowledge to be situated in the current operational environment, as tasks and priorities might vary in relation to operational demands (Ben-Asher and Gonzalez, 2015). In the military context, merging operational demands with the technical aspects of cyber operations results in a need to distinguish cybersecurity from cyber operations (Lathrop et al., 2016). Cybersecurity is concerned with defending own assets; defined as a protected organizational resource (Whitman and Mattord, 2012). In military cyber operations, the focus is: “…*defending* cyber- and cyber-physical systems from known or unknown adversaries and, when authorized, conducting *offensive* cyberspace operations to achieve military objectives” (Lathrop et al., 2016, p 283). Military cyber operators therefore distinguish themselves from civilian cybersecurity operators by using the cyber domain as a utility to create military effects. In addition, they defend and protect own critical assets in order to sustain the ability to deliver military kinetic effects. We argue that cyber operators are not limited to working in the cyber domain, but work in a hybrid environment where cyber, physical aspects, and cognitive effects are interconnected and intertwined. This argument implies that military cyber operators need to be aware of and understand the sociotechnical system, defined as: “...taking both social factors and technological factors into consideration” (Coghlan and Brydon-Miller, 2014, p.720), they are a part of. These task demands alongside high information load, result in cyber operator work to be described as safety-critical (Buchler et al., 2018; Knox B. et al., 2018), cognitively demanding (D’Amico et al., 2016), and require cognitive agility to traverse and maneuver across cyber-physical and tactical-strategic dimensions in order to make sense of their work environment (Jøsok et al., 2016).

**FIGURE 1:**
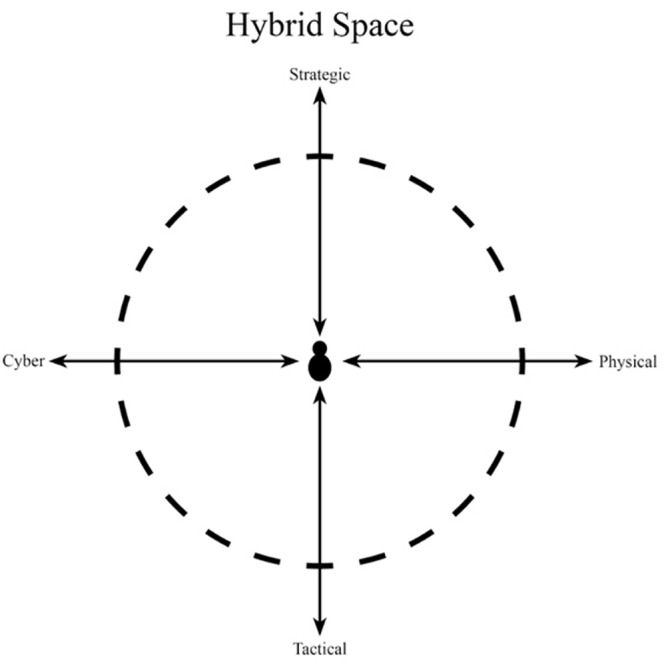
The Hybrid Space conceptual framework (Jøsok et al., 2016).

A recent theoretical proposal (see Figure 1) describes the cognitive work environment of military cyber operators and defined it as *“The Hybrid Space”* conceptual framework (Jøsok et al., 2016).

The framework represents a cyber operator’s range of cognition when conducting cyber operations, taking cyber, cyber-physical, and sociotechnical systems into account. The Hybrid Space framework can be used to measure cyber operator’s cognitive agility. Cognitive agility requires exercise of cognitive focus, which can be understood as an aspect of attention that involves bringing selected information into conscious awareness (MacKay-Brandt, 2011). Individual cyber operator cognitive focus, in this research, is represented by a location in The Hybrid Space, e.g., a cyber operator immersed in coding would be cognitively located in the quadrant facing down to the left (see Figure 1). During the course of a cyber operation, the operator would report different cognitive focus depending on the task. The operator would also be obliged to move cognitively inside, and in-between quadrants depending on the operational requirements. For example, the task of contributing to joint operational planning would require the cyber operator to move to the operational level and traverse into physical domain considerations.

Cognitive agility is defined as a construct made up of three components:

• Cognitive flexibility – ability to cognitively control and shift mental sets and overcome automatic or dominant responses.
• Cognitive openness – being receptive to new ideas, experience, and perspectives.
• Focused attention – ability to attend to relevant stimuli and ignore distracting ones (Good and Yeganeh, 2012).

In line with the above definition, cyber operator capability of cognitive movement by the use of flexible attention and self-regulatory strategies is previously described as displaying cognitive agility (Jøsok et al., 2018; Knox B. et al., 2018) and operationalized as movements (total distance traveled, *x*- and *y*-movement, and quadrant changes) in The Hybrid Space (Knox et al., 2017). Cognitive agility has previously been associated with performance in cyber operations, with higher values of cognitive agility associated with higher level of performance (Knox B. et al., 2018).

Performing deliberate cognitive movements in The Hybrid Space requires observation of and control of own thoughts and actions. Self-regulation refers to the self’s ability to control its own thoughts, emotions, and actions (Baumeister et al., 1994). Self-regulation has previously been linked to individual performance across multiple domains, working through the sustained effort of self-observing behavior, self-directed actions, and performing self-reactive influence (Jaramillo et al., 2017). A large body of studies have linked the ability to self-regulate to positive outcomes in academic achievement and learning in children (Bohlmann and Downer, 2016; Montroy et al., 2016), adolescents (Duckworth and Seligman, 2005; Lerner et al., 2011; Cetin, 2015), and adults (Lerner et al., 2011). Ability to self-regulate has also been linked to development of multiple literacies (Bohlmann and Downer, 2016). Self-regulation is thought to be a relatively stable trait (Shoda et al., 1990), but can be developed through external influence (e.g., modeling and/or mentoring) and own effort (Bandura, 1986). Self-regulation is a well-established and powerful concept that (a) offers the possibility to be measured reliably, (b) can be made subject to training or selection, and (c) is also relevant as it – if shown relevant – might open the opportunity to be used in training of cyber personnel to make better use of their self-regulatory resources. Self-regulation should therefore be explored in relation to displays of cognitive agility and performance in cyber operations. A challenge that remains is establishing consensus of how to assess operator performance in cyber operations (Forsythe et al., 2013). Previous research points to agility and flexible cognitive strategies as pathways to performance in cyber operations (Knox B. et al., 2018). However, how cyber operators maneuver cognitively to make sense of the hybrid environment is unknown. This article explores the relationship between self-regulation, cognitive agility, and performance.

Examining cyber operators in a naturalistic environment, such as during cyber defense exercises, is essential to understanding how they think and work together to conduct successful cyber operations. Few studies have addressed the cognitive competencies of cyber operators, and how these contribute to performance. Our approach seeks to identify individual cognitive competencies that support performance in cyber operators across the hybrid space they are expected to manage. Identification of cognitive competencies that support performance in cyber operators can help develop cyber operator education and training, and pave the way for more focused research in cyber specific competency requirements. As well as advancing the development of reliable performance measures in cyber operations.

## Materials and Methods

### Description of Participants

The participants in this study were cadets attending the Norwegian Defence Cyber Academy (NDCA). This is a military academy organized under the Norwegian Defence University College. The education offered by the NDCA is a 3.5-year study program, where approximately 40 students are recruited every year. Upon successful completion of the program, students are awarded a bachelor’s degree in computer engineering and military studies. Students accepted for this education undergo an officer candidate selection process similar to other military academies in Norway, but with additional demands in science, technology, engineering, and mathematics (STEM) subjects. During selection, cyber-domain specific abilities, motivation, and interest are subject to assessment, as well as health and physical performance. This specific process of selection results in considerable homogeneity in the student group on numerous measures. The subsequent computer and information systems (CIS) and cyber focused education results in knowledge of cyber domain characteristics and understanding of multi-domain military operations. In addition, a mandatory leadership development program is included in the education. The students can therefore be expected to have knowledge and competence in basic psychological and leadership theories (see Knox B. et al., 2018 for a description of the curriculum and pedagogy). In their final year, they are required to take part in a military exercise, named Cyber Defence Exercise (CDX). The CDX marks the completion of the education, and serves as the experimental environment for this study. Participants in the study comprised of 25 cyber cadets (two were removed in the data analysis due to incomplete data sets making the total number of participants *N* = 23), *M*_age_ = 22.7 years, *SD* = 0.71. Students were invited to participate in the research during the preparation week leading up to the CDX. At this time, they were provided all necessary information regarding The Hybrid Space conceptual framework and assessment of own cognitive location in relation to this (Jøsok et al., 2018).

### Experimental Conditions

This study took place in the CDX of November 2017. The purpose of the CDX it to produce a naturalistic environment in which participants have to exercise a variety of competencies in cyber, physical, and social domains in order to excel in proficiency and understanding of interactions occurring in cyber operations. The design of the exercise simulates a real-world scenario, and includes an attacker team, mentors, and an exercise control (EXCON) that manages the cyber-physical training infrastructure. The exercise is driven by an interconnected cyber-physical scenario with the aim of mirroring the complexity of real-life military cyber operations. Using a scenario-based approach allowed students the opportunity to understand the complexity, uncertainty, and interconnectivity associated with a geopolitical multi-domain conflict. Having a real-world scenario with dynamic attacking strategies was expected to create a learning environment in which students lift their head out of their computer and think critically concerning their actions in a broader context. Scenario injects were delivered to participating teams via an EXCON using various means (e-mail, news articles, webpages, etc.) and guided by a comprehensive scenario handbook.

**FIGURE 2:**
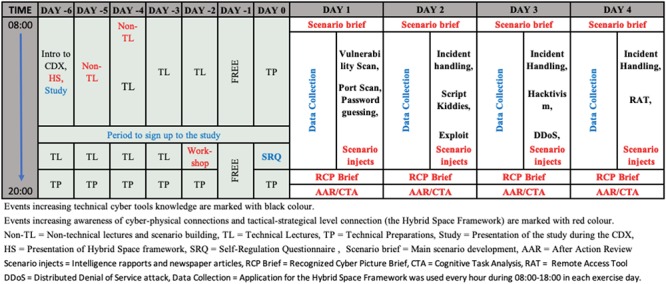
Outline of the different components of the study.

The outline of the components of the study is shown in Figure 2. Students were introduced to the CDX, The Hybrid Space, and the study on the first day (day 6). The following days leading up to the start (6 to 2) were dedicated to technical lectures (TL), non-technical lectures (non-TLs), and technical preparations (TPs). Mentors facilitated a non-technical workshop where students considered different attack scenarios: what could be targeted, who could be behind, the scale and impact for own operations, and how to handle the situation. Students signed up to the study during these days. Self-regulation questionnaires (SRQs) were administered to the participants at day 0. Cognitive agility data were collected from day 1 to day 4 while the students defended their network from the different attacks shown in Figure 2.

The attacker team included three cyber security professionals. The role of attacker team was to attack targets in the infrastructure of the defender team. The attacker team attempted to gain access to data and services, such as websites and e-mails, on the defender team’s networks without being detected. Attack types such as port-scanning, distributed denial of service (DDoS), and remote access tool (RAT) attacks were used. The attacks were synchronized with the existing and ongoing developments in the physical scenario simulation. The scale and sophistication of the attacks progressively increased throughout the exercise.

During the CDX, students were divided into four teams of approximately 10 students and operated as independent security operation centers (SOCs) with the task of defending a network. The role of defender team was to detect and defend against the attacker team attacks while maintaining their normal network services. The groups in the defender team were expected to be pro-active and monitor their network based on their overall situation awareness. The groups were allowed to make decisions themselves relating to the organizational structure (i.e., organizing the responsibilities within the group, such as picking a team leader), the physical structure (i.e., workstation arrangements, display of different maps, and graphical representations), and planning and discussion activities (i.e., providing status updates in team meetings).

The CDX was led by an EXCON team that included external mentors, commander in chief, and subject matter experts (SMEs). The role of the EXCON team was to manage the exercise, play the scenario, host the network infrastructure, coordinate and provide the defender team with necessary inputs to ensure the exercise was executed as intended, and record all network traffic. The external mentors were computer network defense (CND) professionals who were responsible for observing and providing guidance to the SOCs. The mentors were not allowed to directly influence the actions of SOCs, but were allowed to clarify various uncertainties about what to do, and ensure that the SOCs received useful and constructive feedback. The commander in chief was a professional officer. His role was in the physical domain. He acted as the senior ranking officer whose decisions making (e.g., deploying troops on the ground) was dependent upon on situational awareness presented by the SOCs. SMEs were responsible for scenario and story line development and the logic behind them. During the exercise, they made adjustments to the scenario in an effort to ensure that students obtain maximal benefits from such experiences.

### Experimental Infrastructure

A cyber-range was set up with physical hardware and a virtual environment consisting of virtual computers and network equipment. All SOCs had the same/similar hardware, similar physical working conditions, followed the same time-table, and were exposed to the same demands (i.e., ordered to brief the commander in chief, called in to status meetings and delivering the same products based on their current understanding of the situation).

### Data Collection

The SRQ was used to evaluate self-regulatory ability through self-report (Brown et al., 1999). The seven-step model of self-regulation was initially developed to study addictive behavior. However, the self-regulatory processes described in the model are considered to reflect general principles of behavioral self-regulation, the reliability appears to be excellent, and the total SRQ score has been validated to reflect self-regulatory functioning (Miller and Brown, 1991; Brown, 1998). In the SRQ model, behavioral self-regulation is seen as a process and therefore may fault as a result of failure in completing any of these seven steps (Brown et al., 1999):

1. Receiving relevant information
2. Evaluating the information and comparing it to norms
3. Triggering change
4. Searching for options
5. Formulating a plan
6. Implementing the plan
7. Assessing the plan’s effectiveness (which recycles to steps 1 and 2).

A sample item includes “I have personal standards, and try to live up to them” and “When I’m trying to change something, I pay a lot of attention to how I’m doing.” The form has previously demonstrated high internal consistency and reliability (Cronbach’s α = 0.91) and showed acceptable reliability score for this study (Cronbach’s α = 0.75). The SRQ consists of 63 items, and each point is scored through a five-point Likert scale (1 – strongly disagree, 2 – disagree, 3 – uncertain or unsure, 4 – agree, 5 – strongly agree) (Brown et al., 1999). Participants filled out the SRQ prior to the CDX exercise. The items comprise a total score and a score for each subscale.

### Application of the Hybrid Space Framework

**FIGURE 3:**
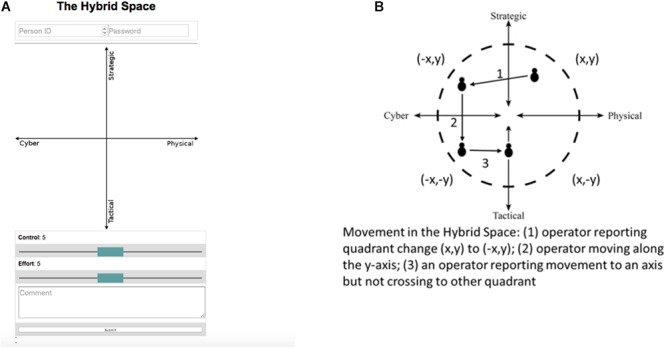
**(A)** Screenshot of The Hybrid Space app. **(B)** Visualization of computed variables.

Cognitive agility data were collected by use of a web-based application where 0 is the center, *X*- and *Y*-axis range from -100 to +100 (see Figure 3A). The application was specifically designed and developed to collect data during the CDX (see Jøsok et al., 2018 for details on the development and application of the data collection app). Students participating in the research were instructed to mark their cognitive location every hour (0800–1800) for 4 consecutive days while participating in the CDX. Students first entered their location in The Hybrid Space (e.g., when conducting malware analysis, one would typically mark a lower left position, and when collaborating in their team making sense of the malware one would typically mark a position lower and to the right based on their human-to-human interaction). When sense making on operational/strategic impact of their findings, one would consider information that required cognitive positioning toward the higher dimensions of The Hybrid Space). Students then entered their perceived level of control and their perceived level of cognitive effort at the moment by adjusting the sliders to a nine-point Likert scale with distinct points ranging from 1 till 9, where 1 represents the lowest subjective assessed momentary effort or control and 9 is the highest level of momentary control or effort. Comments were made voluntary in order to minimize intervention time; however, if they chose to use the comment field, they were instructed to disclose the current task they were engaged in.

**FIGURE 4:**
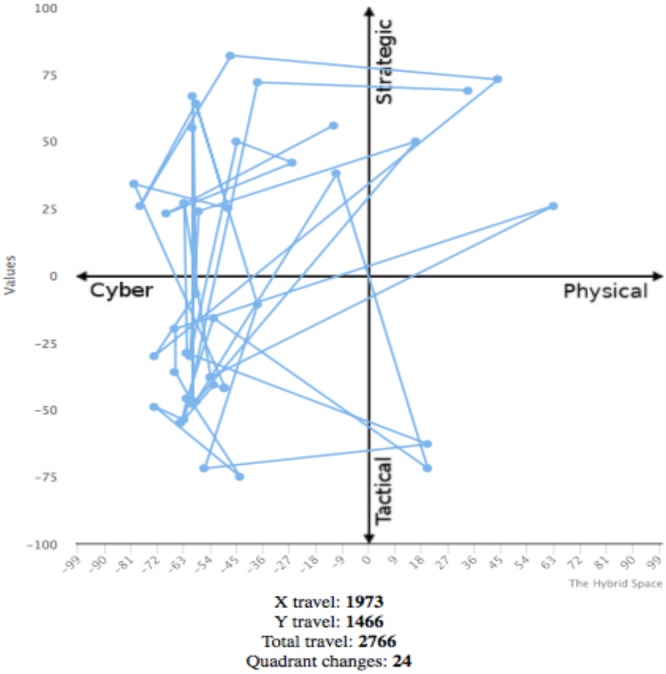
Visualized data from The Hybrid Space App.

For the purpose of analysis, and based on the possible operator reported movements shown in Figure 3B, totals for the following dependent variables were computed; HSDT: total distance traveled in the Cartesian plane measured by Euclidean distance; HSQC: number of quadrant changes; HSxM: movement along the cyber-physical domain (*x*-axis); HSyM: movement along the strategic-tactical domain (*y*-axis). The dependent variables were first developed and reported in Knox et al. (2017). An example of raw data collected from one individual is shown in Figure 4.

### Data Reduction and Analysis

All variables were checked for distribution and normalized if needed. Statistical analysis was then performed with all variables. Correlations and regression analysis were then performed with self-regulation entered as the independent variable and Hybrid Space movements (HSDT, HSQC, HSxM, HSyM) entered as dependent variables. The alpha levels for testing the hypothesis were set at the 0.05 level. All analyses are performed using SPSS v24. Although Cohen’s convention is often used to interpret effect size in psychology (Cohen, 2003), due to a moderate sample size in this pilot study, we have applied a more restrictive wording in accordance with Mukaka (2012) to interpret the effect size of the correlation coefficient. The applied wording is shown in Table 1.

**Table 1:** Interpretation size of correlation coefficient (Mukaka, 2012).

Size of correlation	Interpretation
0.90 to 1.00 (-0.90 to -1.00)	Very high positive (negative) correlation
0.70 to 0.90 (-0.70 to -0.90)	High positive (negative) correlation
0.50 to 0.70 (-0.50 to -0.70)	Moderate positive (negative) correlation
0.30 to 0.50 (-0.30 to -0.50)	Low positive (negative) correlation
0.00 to 0.30 (-0.00 to -0.30)	Negligible correlation

### Ethics Statement

Prior to the start of the exercise, all participants were informed about the overall scope of the study and how to use the Hybrid Space application. Participants signed informed consent prior to the intervention, and were informed of the unquestioned opportunity to withdraw at any time. The project is registered with the Norwegian Social Science Data Services (NSD) project number 55446.

## Results

The comment field (see Figure 3A) was rarely used by the participants, and hence it was excluded in further analysis. Participants also reported their perceived momentary level of effort and control at the same time as entering their cognitive location in The Hybrid Space. However, during analysis, it was decided to exclude the data from this paper in order focus on cognitive agility and self-regulation. Henceforth, the remaining data presented are SRQ data and cognitive agility data. Descriptive statistics are presented in Table 2.

**Table 2:** Descriptive statistics (*N* = 23).

	Mean	Std. deviation	Minimum	Maximum
Cognitive agility HSDT	2225.09	93.71	723	4161
HSQC	17.39	6.92	6	30
HSxM	1539.17	740.41	456	3145
HSyM	1271.96	550.90	446	2595
SRQ SR_Receiving	30.53	4.32	23	38
SR_Evaluating	29.33	4.14	21	41
SR_Triggering	30.41	2.65	26	35
SR_Searching	32.28	3.01	25	36
SR_Planning	31.39	3.99	24	36
SR_Implementing	31.00	4.43	24	38
SR_Assessing	31.00	2.48	26	34
SR_Total	214.33	12.6	199	236

*HSDT: distance traveled in the Cartesian plane measured by Euclidian distance; HSQC: number of quadrant changes; HSxM: movement along the cyber-physical domain (*x*-axis); HSyM: movement along the strategic-tactical domain (*y*-axis); SRQ: self-regulation questionnaire*.

The relationship between cognitive agility (as measured by The Hybrid Space application) and self-regulation (as measured by the SRQ) was investigated using Pearson product-moment correlation coefficient (see Table 3). Preliminary analyses were performed to ensure no violation of the assumptions of normality, linearity and homoscedasticity. Using Mukaka’s (2012) standards for interpreting correlations, all measures of cognitive agility were low to moderately positive correlated to total self-regulation score (SR_total) (see Table 3).

**Table 3:** Pearson’s correlations (*N* = 23).

	2	3	4	5	6	7	8	9	10	11	12
1.HSDT	**0.858^∗∗^**	**0.946^∗∗^**	**0.874^∗∗^**	**0.778^∗∗^**	**0.491^∗^**	**-0.661^∗∗^**	-0.043	0.403	0.260	0.459	**0.685^∗∗^**
r (Clmax-Clmin)	0.938–0.69	0.977–0.875	0.945–0.722	0.901–0.539	0.751–0.099	-0.342–0.843	0.375–0.447	0.699–0.011	0.607–0.17	0.732–0.058	0.855–0.381
2.HSQC		**0.766^∗∗^**	**0.809^∗∗^**	**0.617^∗∗^**	**0.427^∗^**	**-0.502^∗^**	-0.173	**0.448^∗^**	0.347	0.107	**0.543^∗^**
r (Clmax-Clmin)		0.851–0.366	0.915–0.596	0.82–0.275	0.713–0.018	-0.114–0.757	0.257–0.546	0.726–0.044	0.664–0.076	0.497–0.319	0.78–0.169
3. HSxM			**0.676^∗∗^**	**0.812^∗∗^**	**0.436^∗^**	**-0.660^∗∗^**	-0.009	0.362	0.240	0.488	**0.675^∗∗^**
r (Clmax-Clmin)			0.851–0.366	0.917–0.601	0.718–0.036	-0.341–0.214	0.404–0.419	0.673–0.059	0.593–0.191	0.749–0.095	0.85–0.365
4. HSyM				**0.603^∗∗^**	**0.461^∗^**	**-0.526^∗^**	-0.053	0.399	0.273	0.328	**0.588^∗∗^**
r (Clmax-Clmin)				0.813–0.254	0.733–0.061	-0.146–0.771	0.367–0.45	0.696–0.015	0.615–0.156	0.652–0.097	0.805–0.233
5. SR_Receiving					**0.433^∗^**	**-0.470^∗^**	-0.183	0.286	0.220	0.474	**0.740^∗∗^**
r (Clmax-Clmin)					0.717–0.026	-0.072–0.739	0.247–0.553	0.624–0.143	0.579–0.211	0.741–0.077	0.882–0.472
6. SR_Evaluating						-0.196	-0.334	0.020	-0.083	0.178	0.385
r (Clmax-Clmin)						0.235–0.562	0.09–0.655	0.428–0.395	0.34–0.478	0.549–0.252	0.688–0.032
7. SR_Triggering							0.037	-0.190	-0.344	0.158	-0.223
r (Clmax-Clmin)							0.442–0.381	0.241–0.558	0.079–0.662	0.535–0.271	0.208–0.581
8. SR_Searching								-0.014	0.225	0.244	0.181
r (Clmax-Clmin)								0.4–0.423	0.583–0.206	0.596–0.187	0.552–0.249
9. SR_Planning									**0.449^∗^**	0.344	**0.608^∗∗^**
r (Clmax-Clmin)									0.726–0.046	0.662–0.079	0.815–0.262
10. SR_Implement										0.094	**0.529^∗^**
r (Clmax-Clmin)										0.487–0.331	0.772–0.15
11. SR_Assessing											**0.703^∗∗^**
r (Clmax-Clmin)											0.864–0.41
12. SR_Total											1.000
r (Clmax-Clmin)											1–0.998

^∗∗^*Correlation is significant at the 0.01 level (one-tailed). ^∗^Correlation is significant at the 0.05 level (one-tailed). Upper confidence and lower confidence intervals r(Clmax-Clmin) are shown at the 0.05 level. Bold values are both significant at the 0.01 and 0.05 level*.

**FIGURE 5:**
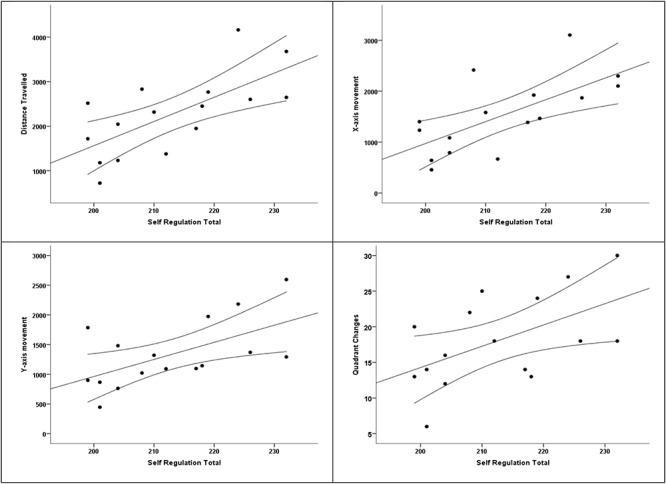
Scatterplots.

Linear regression was used to assess the ability of self-regulation to predict cognitive agility (see Table 4). Computed cognitive agility indicators were set at as dependent variables, and self-regulation total scores were set as independent variable. All self-regulation variables moderately predicted HS movements (see Table 4 and Figure 5). Using this model, self-regulation explained 43.1% of cognitive agility in The Hybrid Space. Looking at the subcomponent of the total movement, self-regulation explained 41.6% of the *x*-axis movement, and 29.9% of the *y*-axis movement; 24.4% of the quadrant changes is explained by self-regulation.

**Table 4:** Regressions for self-regulation and cognitive agility indicators.

Model	*R*	*R*^2^	Adj *R*^2^	*F*	*p*	β	*t*
HSDT	0.685	0.469	0.431	12.372	0.003	0.685	3.517
HSQC	0.543	0.294	0.244	5.843	0.030	0.543	2.417
HSxM	0.675	0.455	0.416	11.692	0.004	0.675	3.419
HSyM	0.588	0.345	0.299	7.384	0.017	0.588	2.717

*HSDT: hybrid space distance traveled; HSQC: hybrid space quadrant changes; HSxM: hybrid space x-axis movement; HSyM: hybrid space *y*-axis movement.*

Scatterplots of the results visualize a moderate positive relationship between higher levels of self-regulation and increased cognitive agility by all variables. Curved lines show confidence intervals to the mean at the 0.05 level.

In summary, display of cognitive agility in The Hybrid Space appears to be predicted by self-regulation when performing defensive cyber operations during this CDX.

## Discussion

This study tested if self-regulation could predict performance of cyber operators during a CDX. The results show that higher levels of self-regulation in cyber cadets are associated with displays of cognitive agility as measured by movement in The Hybrid Space, thus supporting the hypothesis. The environment that this CDX is replicating is earlier described as hybrid (Jøsok et al., 2016), and characterized by novel task demands (McClain et al., 2015), cognitive intense work (D’Amico et al., 2016), challenging situational awareness (D’Amico et al., 2005), team collaboration and coordination perspectives (Champion et al., 2012; Jøsok et al., 2017), communication challenges (Knox B.J. et al., 2018), and challenges in assessing performance (Ben-Asher and Gonzalez, 2015). A cyber operations environment is argued to crave constant adaptation to complexity by cyber operators (Lathrop et al., 2016). This involves displays of higher order cognitive skills (Knox B. et al., 2018) associated with displays of cognitive agility (Knox et al., 2017), here represented by ability to flexibly adjust attention, exercise cognitive control, shift cognitive focus, and regulate responses in The Hybrid Space. Self-regulation has shown similar results in previous studies, suggesting that self-regulation is associated with displays of cognitive agility and performance of cyber operators (Knox et al., 2017; Knox B. et al., 2018). The subcomponents of self-regulation in relation to cognitive agility are discussed below.

Higher levels of self-regulation were associated with more active search for information in The Hybrid Space, meaning that the individual operator traversed cyber and physical domains cognitively, as well as strategic and tactical considerations when seeking out relevant information. As self-observation is a prerequisite for self-regulation (Bandura, 1986), contextual overview of the environment is necessary to situate oneself and one’s actions in The Hybrid Space. Hence, a presupposition for self-regulation action would be to locate oneself and identify human or digital artifacts in the Hybrid Space. Therefore, a behavior that displays high levels of cognitive agility when searching for information in order to make sense of the evolving situation could be considered a performance strategy in cyber operations as this would facilitate better cyber situational awareness (D’Amico et al., 2005). This is supported by the findings that self-regulation receiving behavior was moderately associated with all cognitive agility measurements.

Evaluating the accuracy and importance of the obtained information from one Hybrid Space dimension might require additional revisiting of other locations in The Hybrid Space. This can be the result of a rapid changing situation or that the task challenges limitations in working memory capacity, and requires additional refreshing or confirmation of information. Other explanations can be that operating in change and novelty shifts the demands from problem solving to problem identifying, resulting in needs to continually shift in between searching and evaluating information, at least until an abnormality, challenge, or problem is identified. Prior research confirms that ambiguous shifting conditions require competencies at identifying problems (Lathrop et al., 2016), and that flexible cognitive strategies need to be applied to construct higher levels of understanding of the problem-solving at hand (Ward et al., 2013). Spending effort in this phase makes sense also in a cyber defense setting where a lot of the time nothing happens. A resulting effect may be sustained attention toward understanding the state of affairs as they are, leading to effort that might build proficiency in detecting and evaluating anomalies as they occur. The association between cognitive agility and the self-regulation evaluating subscale is therefore quite possibly interlinked, as searching and evaluating information in cyber operations is a twofold process.

The self-regulation triggering subscale is negatively associated with cognitive agility, and could be interpreted as reduction in distance covered in The Hybrid Space. This might be a natural consequence of the two prior subfunctions, searching and evaluating, as a stop/temporary pause in Hybrid Space probably can be triggered by identifying information that requires closer scrutiny. For example, if a piece of code or a specific internet protocol (IP) address requires attention, this would temporarily limit the need for searching.

Self-regulation in planning, implementation, and evaluating shows low positive association with cognitive agility. However, the variations between planning and implementing are interesting. While planning shows low to moderate association, implementing shows in general low association. Planning might require the cyber operator to zoom out of the current focus in The Hybrid Space and engage in conversations with the team in order to share understanding and come up with ideas to tackle the problem identified. In this vain, a cyber operator might traverse the cyber, physical, and social domains in an operational planning process, producing high levels of cognitive agility. Further, when a solution (or in the absence of a solution) is present, implementing would not necessarily require high levels of cognitive agility as the solution might be limited to implementation in one part of quadrant of The Hybrid Space. Lastly, assessing the impact of the implementation shows low to moderate correlation with cognitive agility. This might be explained by the process of self-regulation which at this stage will return to stage one and two (searching and evaluating) (Brown et al., 1999).

According to the regression models, a total of 43.1% of the cognitive agility in The Hybrid Space can be explained by the self-reported trait of self-regulation. In an applied setting, a lot of contributing factors are at play. Team dynamics are previously shown to influence operator behavior (Champion et al., 2012) and team performance (Buchler et al., 2018), and could both boost or limit individual movement in The Hybrid Space, depending upon high or low team cohesion. Expert mentors triggering movement by asking questions or observing operator performance can also explain movement in the Hybrid Space. The research itself can produce a Hawthorne effect by introduction of The Hybrid Space conceptual framework and instructing participants to mark their cognitive location, constantly nudging operators to reflect over their current cognitive location. Despite the uncertainties addressed, we consider this as relatively strong results when accounting for the naturalistic setting of the CDX, the applied research approach and the novelty of The Hybrid Space approach. As self-regulation had moderate to high positive association with all Hybrid Space movements, the results state that The Hybrid Space can be used to assess levels of self-regulation and the display of cognitive agility among cyber operators.

With the self-regulation construct being linked to performance in a variety of domains, and especially important for learning, it is likely that cognitive agility in The Hybrid Space can be closely linked to performance. High levels of self-regulation have been associated with sticking to behaviors consistent with long-term goals (Brown et al., 1999), and in the context of military cyber operator tasks this implies ability to make decisions regarding in the moment activity that is consistent with reaching overall operational goals. This means that the cyber operator has to have understanding of the overall operational goals as well as own tactical goals and how actions in the cyber domain might influence both. Cognitive agility in The Hybrid Space could support the individual cyber operator to perform better by taking actions in line with the overall context by enabling better contextual knowledge and understanding. However, there is to date no consensus about the operationalization, the assessment, and the quantification of cyber operator performance (Mancuso et al., 2014; Lathrop et al., 2016). There are though attempts to understand performance by comparing the use of software tools between novices and experts (McClain et al., 2015). With the current difficulties in assessing performance in cyber operations, and the absence of performance indicators in cyber operations, the proposed causality between displays of cognitive agility and performance can serve as a pathway to further research and insight into human performance in cyber operations. Building on previous research results proposed in Jøsok et al. (2016) and Knox et al. (2017), we see this as a step further in validating The Hybrid Space as a not only a conceptual model, but also as a tool for assessing individual performance in cyber operations.

This research was approached as a naturalistic and descriptive study in an applied setting, and as such correlational in nature. Further systematic research is needed in which causal pathways are identified, and the complex concepts of self-regulation and cognitive agility investigated in more detail, including intervention studies on enhancement of these skills in cyber operator education. In order to confirm the findings in this study, larger samples are required, as well as developed performance measures to assess levels of cyber operator performance.

## Conclusion

The results support the hypothesis by showing that self-regulation predicts cognitive agility in cyber operators, as measured by cognitive focus movements in The Hybrid Space conceptual framework, when performing defensive cyber operations during a CDX. Theories of cyber operator competencies highlight that cyber operators need a varied skill-set and competencies beyond technical proficiency to perform well; previous research has associated cognitive agility to performance in cyber operations. Our results are in line with theories of cyber operator competencies, and we contribute to cyber operator competence profiles by confirming that cyber operators’ self-regulation is associated with performance in cyber operations, in a training environment. This work highlights the need to focus on developing cyber operators soft skills as pathways to better performance. Future work should include investigating cognitive agility in relation to reliable performance measures in cyber operations to evaluate the association between cognitive agility and performance in cyber operations.

## Ethics Statement

The project is approved by Norwegian Centre for Research Data with project number: 55446 and project title: Grow up digital – Developing cognitive agility and decision-making competence to maneuver in domains of complexity. The following information sheet was distributed, read, and signed by each participant prior to the data collection.

## Author Contributions

ØJ contributed to the ideas, design, preparation, and execution of the study as well as the analyses of results, drafting, necessary theory research, and write up of all parts of the manuscript. RL contributed to data preparation, data analyses, writing of results, as well as writing the manuscript. BK contributed to designing, planning, and execution of the CDX as well as execution of the study, interpreting results, and improving the manuscript. KH contributed to improving the manuscript. SS contributed to framing the manuscript, interpreting the results, and improving the manuscript.

## Conflict of Interest Statement

Three of the authors were employed at the NDCA at the time of data collection. The remaining authors declare that the research was conducted in the absence of any commercial or financial relationships that could be construed as a potential conflict of interest.

## Footnotes

1 According to Collins dictionary (2018), soft skills are defined as: “…interpersonal skills such as the ability to communicate well with other people and to work in a team."

2 Wang (1990) describes cognitive competency as: “a psychological construct that cannot be directly observed but can be inferred from an individual’s behaviour or performance on content-relevant tasks" (p. 219).
